# Harnessing Biomaterials to Engineer the Lymph Node Microenvironment for Immunity or Tolerance

**DOI:** 10.1208/s12248-014-9708-2

**Published:** 2014-12-23

**Authors:** James I. Andorko, Krystina L. Hess, Christopher M. Jewell

**Affiliations:** 1 Fischell Department of Bioengineering, University of Maryland, 2212 Jeong H. Kim Engineering Building, College Park, Maryland 20742 USA; 2 Department of Microbiology and Immunology, University of Maryland Medical School, Baltimore, Maryland USA; 3 Marlene and Stewart Greenebaum Cancer Center, Baltimore, Maryland USA

**Keywords:** autoimmunity and tolerance, biomaterials, immunology, nanoparticles and microparticles, vaccine

## Abstract

Nanoparticles, microparticles, and other biomaterials are advantageous in vaccination because these materials provide opportunities to modulate specific characteristics of immune responses. This idea of “tuning” immune responses has recently been used to combat infectious diseases and cancer, and to induce tolerance during organ transplants or autoimmune disease. Lymph nodes and other secondary lymphoid organs such as the spleen play crucial roles in determining if and how these responses develop following vaccination or immunotherapy. Thus, by manipulating the local microenvironments within these immunological command centers, the nature of systemic immune response can be controlled. This review provides recent examples that harness the interactions between biomaterials and lymph nodes or other secondary lymphoid organs to generate immunity or promote tolerance. These strategies draw on mechanical properties, surface chemistry, stability, and targeting to alter the interactions of cells, signals, and vaccine components in lymph nodes. While there are still many unanswered questions surrounding how best to design biomaterial-based vaccines to promote specific structures or functions in lymph nodes, features such as controlled release and targeting will help pave the way for the next generation of vaccines and immunotherapies that generate immune responses tuned for specific applications.

## INTRODUCTION

Vaccination has produced one of the greatest impacts on human health in history (1). No other breakthrough has virtually eradicated fatal diseases like polio or small pox with just a few doses. However, many diseases impacting public health create complex challenges for existing vaccine and immunotherapy strategies. For example, HIV evades clearance by mutation and concealment in the mucosa, tumors actively suppress tumor-destructive immune cells, and many treatments for autoimmune disease lack specificity. To address challenges such as these, new vaccines and immunotherapies will need to generate potent responses against specific molecules—termed antigens—while also tuning the characteristics of these responses to combat a target disease. Lymph nodes (LNs) and the spleen are some of the key structures that coordinate the type and specificity of these responses.

In the last several years, the impact of nanoparticles (NPs), microparticles (MPs), and other biomaterial vaccine and immunotherapy carriers on LNs has been an intriguing area of focus. These studies reveal the potential of biomaterials to program the local LN microenvironment to control systemic immune response. The broad potential of biomaterials for vaccination and immunotherapy has recently been reviewed (2–4). This paper focuses more specifically on the interactions of biomaterials with LNs and other immune tissues (e.g., spleen) during the generation of stimulatory or regulatory immune responses. The discussion begins with background describing how adaptive immune responses are generated, with an emphasis on the active role that LN tissues and resident cells play in these processes. Key recent examples are then discussed to demonstrate how biomaterials enhance the generation of immunity, for example, against a foreign pathogen, or of tolerance, such as to combat autoimmune disease. The review concludes by identifying unanswered questions and highlighting some of the ways in which answers to these questions could inform new approaches to exploit the interactions between biomaterials and LNs for vaccination, immunotherapy, and tissue engineering.

## ADAPTIVE IMMUNITY REQUIRES STRUCTURED INTERACTIONS BETWEEN IMMUNE CELLS

### Antigens in Peripheral Tissue Must Reach LNs to Initiate Adaptive Immune Response

The innate immune system is composed of first-response defense mechanisms including (i) skin that creates a physical barrier against pathogens, (ii) immune cells that home to and engulf pathogens or other immunogenic structures, and (iii) receptors that detect broad classes of molecular patterns absent in mammals but present in viruses and bacteria. In contrast, adaptive immunity involves the generation of immune responses specific for a particular molecule, termed an antigen. Generation and control of these antigen-specific responses require complex interactions between immune cells, antigens, and soluble factors in secondary lymphoid organs (SLOs) (5,6). These tissues include the spleen, LNs, and Peyer’s patches. The spleen samples circulating antigens present in blood, while specialized nodules termed Peyer’s patches sample antigens in mucosal tissues such as the small intestine.

**Fig. 1:**
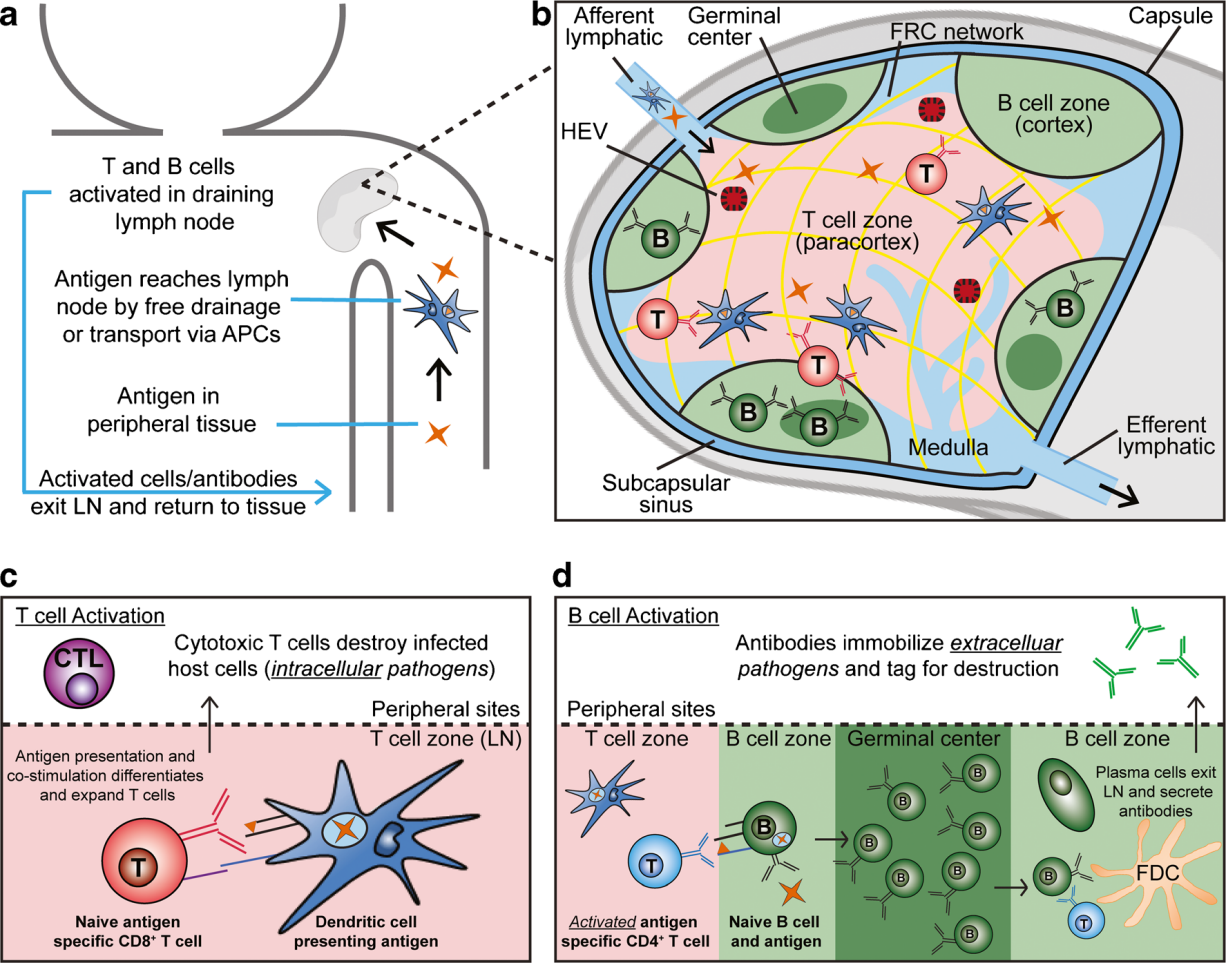
Schematic overview of cell-mediated and antibody-mediated immunity relating to the LN. **a** Graphical depiction of antigen drainage through lymphatics and APC-aided transport to LNs. **b** Illustration of the LN microenvironment containing key cells and stromal structures. **c** Activation of cytotoxic T cell is induced by DCs which process and present antigens with co-stimulatory molecules to naive CD8^+^ T cells within the T cell zones of LNs. **d** B cell activation occurs after an activated CD4^+^ helper T cell binds to B cells presenting the same antigen at the periphery of the LN follicle. Activated B cells then migrate to germinal centers (GCs) where proliferation, somatic mutation, and, with the help of follicular DCs and follicular helper T cells, affinity maturation occur. These processes result in plasma cells that exit the LN and secrete high-affinity antibodies

LNs are found throughout the body, concentrating antigens from a network of lymphatic vessels that continually sample tissue for antigens or other immune signals (7,8). Soluble antigens with molecular weights of ~70 kDa or with particle size between 20 and 50 nm passively drain along the lymphatics, while larger antigens or pathogens are phagocytosed and carried to these LNs by specialized antigen-presenting cells (APCs) such as dendritic cells (DCs) (Fig. 1a) (2,9). APCs continually survey tissue and blood for inflammatory signals and antigens, which upon detection, stimulate phagocytosis and a change in the expression of homing receptors that allows antigen-experienced APCs to travel to nearby “draining” LNs (7). In LNs, processed antigens are presented by APCs to activate resident T and B lymphocytes. Activated lymphocytes and molecules secreted by these cells (e.g., antibodies) exit LNs and search the periphery to immobilize or destroy the pathogens against which they are armed in LNs. Thus, LNs are key structures that vaccines and immunotherapies must reach to generate antigen-specific responses that can combat pathogens and diseased tissue located in other regions of the body.

### LNs Contain Supportive Stromal Components, B Cell Zones, and Regions Rich in APCs and T Cells

LNs are bean-shaped structures surrounded by a collagen-rich fibrous capsule (Fig. 1b). Antigens—in soluble form or phagocytosed within APCs—enter LNs via the afferent lymphatic that drains lymph fluid flowing from upstream lymphatic vessels (7,8). This fluid travels around the periphery via the subcapsular sinus (SCS), a region rich in macrophages able to take up and process incoming antigen or particles, and is dispersed throughout the LN by supportive stromal tissues that include fibroblastic reticular cells (FRCs) and extracellular matrix (ECM) components secreted by FRCs (5). This network of conduits and cells ensures that small, soluble antigens can efficiently penetrate deep into LNs.

DCs and T cells comprise an interior region of LNs called the paracortex (“T cell zone”), while B cells and specialized follicular dendritic cells (FDCs) make up a surrounding cortex called the “B cell zone” (Fig. 1b) (10,11). The degree of intermingling between these two regions is controlled by soluble chemotactic factors called chemokines. The chemokines CCL19 and CCL21 attract T cells expressing CCR7 to the paracortex, while CXCL13 attracts B cells expressing CXCR5 to the follicles of the cortex (10,11). During generation of adaptive immunity, this balance changes: B cells upregulate CCR7 receptors for CCL19/CCL21, while T cells upregulate CXCR5 receptors for CXCL13, promoting interactions between APCs, T cells, and B cells at the interface of the T and B cell zones. The purpose of these interactions is to generate effector cells and secreted antibody molecules specific for a particular antigen encountered in organs, blood, or peripheral tissue. Upon activation and expansion, T cells and B cells are collected and exit the LN through the medulla and efferent lymphatic. Structures called high endothelial venules (HEVs) also connect LNs with circulatory vasculature, serving primarily as a conduit for lymphocytes to travel between blood and LNs. A summary of the key cells and structures of the LN can be found in Table I.

**Table I:** Key Cells and Structures Comprising Lymph Nodes

Cell or tissue	Acronym	Key function
Professional antigen presenting cell	APC	Cells exhibiting a primary function of processing and presenting antigen. Key populations include DCs, B cells, and macrophages.
Dendritic cell	DC	APCs surveying peripheral tissue for antigen. DCs take up antigen, migrate to LNs, then present antigen to T and B cells to generate antigen-specific immunity.
T lymphocyte	T cell	Cells involved in direct cell killing of infected host cells (CD8^+^ cytotoxic T cell population) and helper functions that support antibody production (CD4^+^ helper T cells).
B lymphocyte	B cell	Cells that differentiate to plasma cells that are able to secrete antibody molecules that bind antigens. Binding leads to neutralization or destruction of these targets.
Fibroblastic reticular cell	FRC	Stromal cells that support trafficking of soluble signals and antigen throughout LNs. These cells also organize LN structure by secreting extracellular matrix components.
Follicular dendritic cell	FDC	Specialized dendritic cells able to capture and present antigen to B cells in GCs to promote high-affinity antibodies.
Capsule		Dense layer of connective tissue that surrounds the internal structure of LNs.
Afferent lymphatics		Entry of antigen and immune cells from lymphatics.
Efferent lymphatics		Exit of immune cells from LNs to lymphatics.
Subcapsular sinus	SCS	Drains and distributes lymph throughout LNs.
Medulla		Drains activated lymphocytes in LNs to efferent lymphatics for return to tissue and blood.
High endothelial venule	HEV	Portal allowing exchange of lymphocytes with blood.
T lymphocyte zone (paracortex)		Interior domain rich in T cells and DCs.
B lymphocyte zone (cortex)		Follicular region located at the peripheries of the paracortex that is rich in B cells and FDCs.
Germinal center	GC	Structures that form to co-mingle specialized DCs, helper T cells, and B cells during induction of high-affinity antibodies.

### Adaptive Immunity Requires Specific Interactions Between APCs, T Cells, and B Cells in LNs

The major classes of adaptive responses include i) cell-mediated immunity, through which cytotoxic T lymphocytes directly destroy infected host cells, and ii) antibody-mediated immunity, which involves binding, neutralization, and clearance of antigens by circulating antibodies specific for these pathogens. In the simplest sense, cell-mediated immunity removes intracellular pathogens such as viruses, while antibodies are able to address extracellular toxins and pathogens (e.g., bacteria). Cell-mediated and antibody-mediated responses develop following the activation of naive, antigen-specific T cells and B cells, respectively (12,13). These processes involve interactions with APCs in LNs or other SLOs. Naive CD8^+^ T cells are activated by DCs that have encountered, processed, and are presenting the antigen these T cells are specific for (i.e., a “cognate” antigen) (Fig. 1c). Importantly, this activation requires that the cognate antigen be presented by the APCs in a protein complex called major histocompatibility complex I (MHC-I). Activation also requires co-stimulatory surface molecules that are expressed when DCs encounter inflammatory signals—often adjuvants in the case of vaccines. These agents enable DCs to co-present co-stimulatory signals to CD8^+^ T cells during antigen presentation. This set of interactions causes CD8^+^ T cells to expand and differentiate to cytotoxic T lymphocytes (CTLs) that migrate from LNs to destroy host cells expressing the target antigen (e.g., due to a viral infection). A similar process occurs in DCs presenting antigen in MHC-II to CD4^+^ helper T cells that play an important role in the activation of B cells to produce antibodies.

B cell activation is initiated when B cells in the cortex encounter their cognate antigen, altering the balance of chemokine receptors on these cells and causing migration toward the T cell zone (Fig. 1d). Simultaneously, helper CD4^+^ T cells with the same antigen specificity migrate toward the B cell zone following activation by DCs. B cells are activated at the edge of the cortex by these helper CD4^+^ T cells, then move back into the cortex and proliferate to form a germinal center (GC). In GCs, the affinity of the proliferating B cell for the cognate antigen is increased through interaction with resident FDCs that deliver survival signals to B cells that strongly bind antigens presented by FDCs. These processes involve somatic mutation and affinity maturation and are detailed in recent reviews (14,15). The result of these events is the differentiation of B cells to plasma cells that migrate to the periphery and bone marrow to secrete high-affinity, antigen-specific antibodies that enter blood and peripheral tissue.

### The LN Microenvironment Actively Impacts the Development of Immunity or Tolerance

**Fig. 2:**
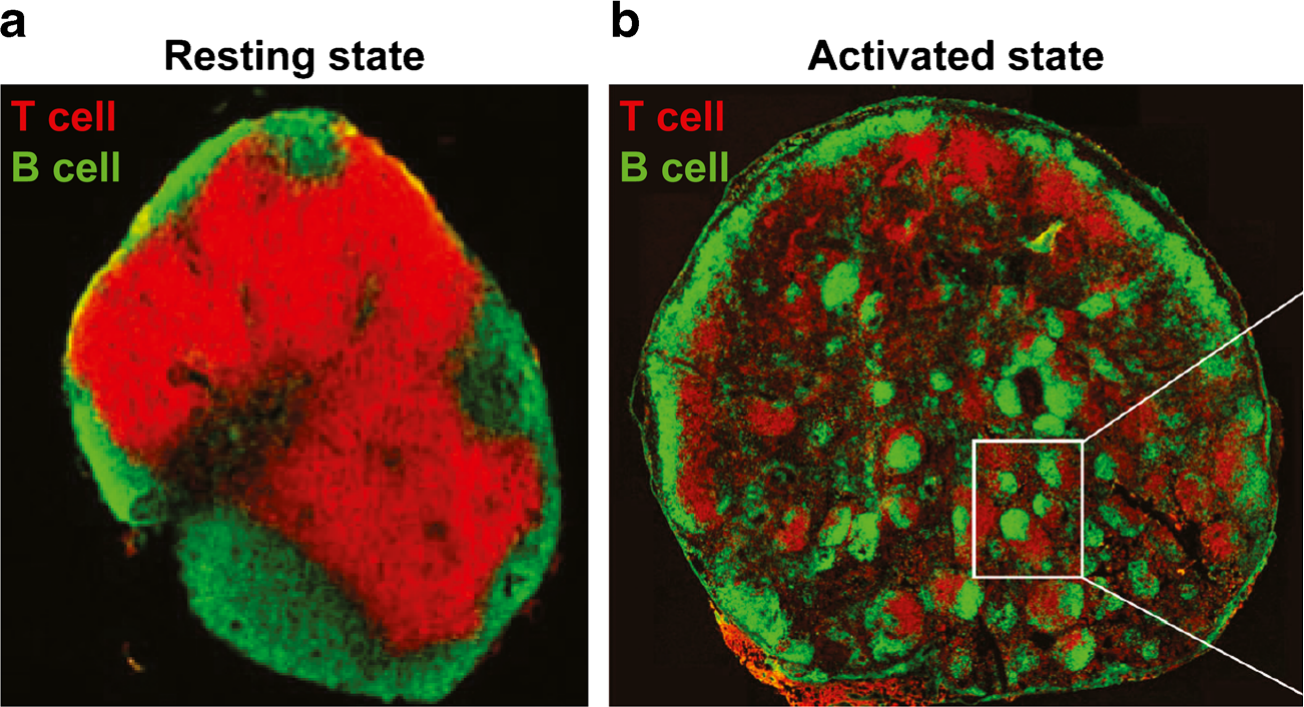
LN reorganization during generation of adaptive immune response. **a** A LN in a resting state with distinct B cell and T cell zones. **b** After activation with antigen and a strong adjuvant (complete Freund’s adjuvant), the LN microenvironment rearranges to promote intermingling of B cell and T cell zones and formation of GCs. Adapted with permission (16)

One of the fascinating developments over the past decade has been the realization that stromal components of LNs and other SLOs not only serve a structural function but also actively promote immunity or tolerance. For example, in the absence of antigen and activating signals, T and B cell zones are maintained in a segregated arrangement (Fig. 2a) (16). In contrast, during generation of adaptive immunity, the LN rearranges to promote specific types and durations of interactions between APCs, T cells, and B cells (Fig. 2b) (16). The FRC network and other stromal components support interactions such as these through production of ECM components, transport of antigens and signaling molecules (e.g., chemokines and cytokines), and establishment of conduits through which lymphocytes travel. Recent studies also illustrate that lymphocytes migrate toward discrete microdomains of LNs during inflammation and immunity compared with migration during tolerance. These effects also correlate with upregulation and downregulation of specific stromal components such as laminins (17). Thus, the combinations of antigens and immune signals present in LNs, along with the specific organization of these tissues, help determine the types of immune responses that develop systemically. Below, we discuss how biomaterials offer new ways to control these parameters to promote stimulatory immune responses (i.e., immunity), as well as to regulate or redirect responses toward immune tolerance.

## BIOMATERIALS EXHIBIT MATERIAL PROPERTIES THAT ACTIVATE IMMUNE PATHWAYS

The physicochemical properties of biomaterials can act as intrinsic immune signals that help shape immunity. Some of the properties which have been studied along these lines—reviewed in (2–4)—include molecular weight, surface chemistry, and particle shape and size. This body of work has demonstrated that biomaterial properties alter lymphatic transport, DC uptake and activation, activation of inflammatory pathways (e.g., toll-like receptors (TLRs) and inflammasomes), and secretion of signaling proteins called cytokines (9,18–31). For example, carriers such as poly(lactic acid-co-glycolic acid) (PLGA) and polystyrene have been shown to activate the immune system through inflammasome signaling even in the absence of other immune signals or adjuvants—agents added to vaccines to enhance immune response (32–34). Since synthetic biomaterials such as PLGA or naturally occurring biomaterials like chitosan are becoming ubiquitous in the design of vaccine and immunotherapy carriers, understanding the link between material properties and immune response could allow more rational design of materials that serve not only as carriers but also as agents that actively “tune” immune responses to combat infectious disease, cancer, or autoimmunity. Thus, this review focuses on the impact of biomaterials on LN organization and function to promote immunity or to regulate immune response (tolerance). Emphasis is also placed on highlighting new approaches reported in the last several years. Table II summarizes the examples presented below that investigate NPs and other biomaterial-based vaccines to help control the structure and function of LNs and other SLOs.

**Table II:** Examples Demonstrating the Impact of Biomaterials on Lymph Nodes

Setting	Biomaterial	Functional impact on lymph nodes	Reference
Immunity	Poly(lactic acid-co-glycolic acid) (PLGA) and polystyrene	Inflammasome activation and increase in IL-1β secretion	(32,33)
Immunity	Chitosan/heparin	Increase in GC formation compared to soluble formulations	(45)
Immunity	Pluronic-stabilized poly(propylene sulfide)	Targeting of tumor-draining LN; delivery of vaccines overcame immunosuppressive tumor environment by activating DCs and increasing the CD8^+^ T cells to T_REG_ ratio	(49,50)
Immunity	Lipid vesicles and micelles	Effective drainage to LN sinuses and increased uptake by APCs leading to increased antigen specificity and cytokine secretion	(28)
Immunity	ICMVs	Trafficking to LN-resident macrophages and DCs in SCS; induces GC formation resulting in long lasting, high-avidity antibodies	(41–43)
Immunity	PLGA	Adjuvant-loaded NPs synergistically increase antibody-mediated immunity through creation of GCs and high-avidity antibodies	(44)
Immunity	Lipid stabilized PLGA	Increased antigen specific CTLs and antibody production caused by local depot effect in LNs	(53,54)
Tolerance	PLGA	Trafficked to LNs; preferential uptake by macrophages and DCs; increases DC activation and CD4^+^ helper T cell proliferation; upregulation of PD-L1; induction of antigen-specific FoxP3^+^ T cells	(4,40,70,84,85,89)
Tolerance	Liposomes	Uptake by LN-resident APCs leading to expansion of T_REGS_ specific for self-antigens included in liposomes	(71)
Tolerance	Iron oxide	Expansion of low-avidity T_REGS_ in and around LNs that suppress antigen presentation by APCs and directly kill APCs	(73)
Tolerance	Polystyrene beads	Support antigen presentation to DCs leading to inactivation of antigen-specific CD4^+^ T-cells; reduction of CD4^+^ and CD45^+^ cell infiltration into CNS; reduction in antigen-specific inflammatory T cell proliferation	(80)
Tolerance	Poly(ethyleneimine)	Trafficking to follicular and marginal zones of LNs; promotes interactions between DCs and T cells with regulatory characteristics	(86)
Tolerance	Poly(ethylene glycol) and poly(lactide)	DCs and T cells modified by particles drain to LNs, reducing the number and proliferative capacity of effector T cells; increase T_REGS_ in LNs; decrease IFN-γ-producing cells	(87–89)

## THE INTERACTIONS OF BIOMATERIALS IN LNs CAN BE EXPLOITED TO ENHANCE IMMUNITY

Biomaterials provide a unique platform for vaccination and immunotherapy. Interestingly, these materials can mimic some features of clinically approved adjuvants (e.g., alum), for example, by condensing or encapsulating antigen or other immune signals into particulate structures with sizes ranging from tens of nanometers to several microns. This size range allows efficient uptake by APCs. Biomaterials can also be used to passively or actively target immune tissues such as LNs. Lastly, these materials allow co-delivery of multiple cargos (e.g., antigen, adjuvant, and drug) and controlled release of vaccine and immunotherapy components. This last feature of co-delivery is becoming increasingly important in modulating the types of responses that are generated for a specific vaccine or therapy. The sections below will provide specific examples of how these properties are being harnessed to enhance “traditional” immune responses aimed at arming the body to destroy infectious pathogens or cancer.

### Particle Size Helps Determine the Trafficking and Retention of Biomaterials in LNs

Both traditional (i.e., soluble) and biomaterial-based vaccine components must reach LNs to generate adaptive immune responses. Several groups have carefully controlled the size of NPs and other vaccine carriers to passively target LNs and the DCs residing in these tissues. For example, by altering particle size, the effectiveness of drainage through the lymphatics and the retention time within LNs can be changed. In studies conducted by Reddy et al., poly(propylene sulfide) NPs with defined sizes were injected intradermally into the tail of mice and the particle drainage through the lymphatics to LNs was monitored (9,35). Twenty- and 45-nm particles drained effectively through lymphatic vessels to the LNs, while 100-nm particles largely remained at the injection site. Additionally, 20-nm particles were preferentially taken up by LN-resident macrophages and DCs. These particles were also retained in LNs for more than 4 days (35). A related study demonstrated that the surface chemistry of NPs altered DC activation and antigen-specific T cell responses in LNs, underscoring the idea that both physical and chemical properties of materials play a role in skewing immune function (9).

The route of injection also helps define if and how NPs of a given size will reach LNs. For example, injection of 90 nm virus-like particles via multiple different injection routes (e.g., subcutaneous, intraperitoneal, intramuscular, and intradermal) resulted in unique particle drainage patterns to the inguinal, lumbar, popliteal, and sciatic LNs (36). Notably, intradermal injections resulted in NPs localized in the SCS of the sciatic and popliteal LNs, supporting the hypothesis that these relatively small diameter NPs were transported via lymphatic vessels to the LNs. As opposed to larger particles trafficked to LNs by APCs, these smaller particles drain freely to the LNs through the afferent lymphatics and can then be scavenged by SCS-resident APCs (e.g., macrophages). Thus, by controlling the injection route and size of NPs or MPs, the domains that these materials reach in LNs can be controlled. This strategy provides a route to design vaccines that specifically target APCs within LNs for phagocytosis or that are small enough to penetrate deeper into other domains (e.g., T cell zone).

Though there are only a handful of approved—or nearly approved—adjuvants in the USA and European Union developed over the last century, in a sense, these agents are the original biomaterial-based vaccine components. One of the most widely used is aluminum salts (alum), and others include emulsions (MF59 and AS03), liposomes (AS01), and synthetic DNA and RNA sequences (polyI:C and CpG). Though there is still some debate as to the mechanism by which alum or other adjuvants enhance vaccination, these materials often persist at the injection site to serve as a depot (i.e., “controlled” antigen release) and increase antigen phagocytosis and presentation that enhances DC activation (37). In a direct comparison of alum with poly(lactic acid) (PLA) MPs, intramuscular immunization with either PLA or alum promoted antigen-specific antibodies. Interestingly, PLA MPs increased expression of MHC-I and MHC-II molecules on DCs, while alum only increased MHC-II expression (34). This finding suggests that particle-based vaccines may generally enhance antigen cross-presentation—a process by which DCs can present phagocytized antigens in MHC-I molecules to enhance CD8^+^ T cell-mediated adaptive response instead of MHC-II molecules which is the traditional route for phagocytized antigens. In addition to particle size, recent studies have investigated the role that the geometry of vaccine carriers plays in immunogenicity. These studies demonstrate that the shape and aspect ratio of synthetic carriers play an important role in modulating T cell activation (29). Thus, future vaccines could combine the rational selection of properties such as size or shape with targeting or controlled release of multiple antigens, adjuvants, or immune signals.

### Molecular Markers Can Be Used to Effectively Target LN-Resident Cells

In addition to passive targeting by size, LNs and LN-resident cells are being actively targeted by conjugating NPs and MPs with specific ligands or receptors. One of the molecules that has been targeted is DEC-205 (CD205), a transmembrane protein found primarily on DCs. Monoclonal antibodies specific to DEC-205 have been used to decorate acid-degradable polymer and liposome vaccines loaded with model antigens (e.g., SIINFEKL from ovalbumin) and B16-melanoma antigens (38,39). Treatment of mice with anti-DEC-205 particles increased the amount of vaccine present in DCs residing in the inguinal LN following subcutaneous immunization and in the popliteal LN following a footpad injection. When administered with LPS or interferon gamma (IFN-γ) to activate DCs, this increase in vaccine accumulation in the LNs correlated to increases in splenic cytotoxic T lymphocytes. Following an intravenous melanoma challenge, the number of tumors in the lungs decreased in mice treated with anti-DEC-205 particles compared to control particles conjugated to an irrelevant targeting peptide. This approach demonstrates the promise of actively targeting biomaterials to specific LN-resident populations to enhance systemic adaptive immune responses.

**Fig. 3:**
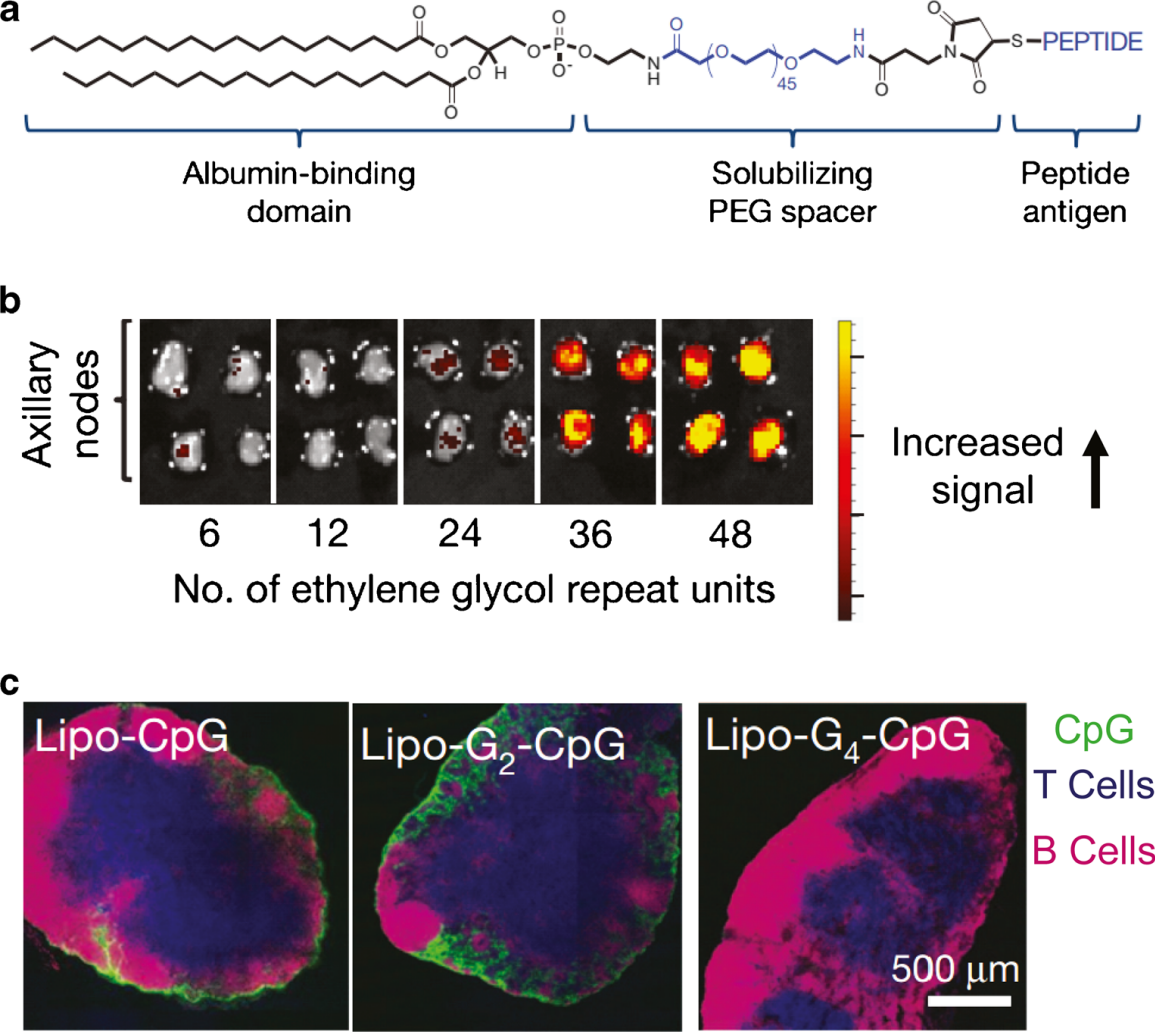
Trafficking of lipid-based vaccines depends on material properties. **a** Schematic of amphiphile structure containing an albumin-binding domain, PEG spacer, and peptide antigen. **b** Excised LNs of mice imaged by IVIS 24 h after treatment with fluorescent amphiphiles with increasing PEG spacer length. **c** Immunohistochemical staining of inguinal LNs following treatment with micelles with varying amounts of guanine repeats. CpG (*green*), T cells (CD3, *blue*), B cells (B220, *pink*). Adapted with permission (28)

Liu et al. recently used albumin as a shuttle to direct lipid-based vaccines to LNs (28). Albumin is a serum protein that serves to transport fatty acids from the blood into lymphatics and to LNs. To exploit this pathway, lipids containing an albumin binding domain made from a diacyl tail were conjugated to peptide antigens and CpG—a TLR9 agonist that activates TLR pathways triggered by non-mammalian DNA (e.g., from bacteria) (Fig. 3a). These materials are able to self-assemble into micelles when placed in aqueous solution due to the hydrophobic diacyl lipid tail. Following subcutaneous injections in mice, albumin-targeted micelles efficiently drained to axillary and inguinal LNs, while formulations with low albumin-binding domains were not effectively trafficked to LNs. Interestingly, by altering the length of a polyethylene glycol (PEG) spacer or increasing the number of carbons in the lipid backbone, vaccine accumulation in the draining LNs could be controlled (Fig. 3b) (28). Mechanistic studies revealed that micelle stability played a crucial role in how these materials were trafficked to LNs. Micelles were stabilized with guanine repeat units. Stabilization with four or more guanine repeats (Lipo-G4-CpG) did not support trafficking of micelles to LNs, whereas reversible (i.e., non-stabilized) micelles assembled with zero or two guanine repeats (Lipo-CpG and Lipo-G2-CpG, respectively) reached LNs and were co-localized with macrophages and DCs (Fig. 3c). The dependence of LN trafficking on structure suggests that in the micelle form, albumin is unable to access the binding domain (diacyl lipid tail), preventing albumin-mediated trafficking to LNs. Building on these findings of increased accumulation and retention time of the albumin-binding micelle vaccines in LNs, peptides specific to HPV-derived cervical cancer or melanoma were added to these structures and used to immunize mice after tumor inoculation. In both of these disease settings, a striking increase in antigen-specific CD8^+^ T cells and functional inflammatory cytokines (IFN-γ and tumor necrosis factor alpha (TNF-α)) was observed, resulting in tumor regression and prolonged survival in immunized mice. These highly promising outcomes are fundamentally a result of the higher concentrations (targeting) and retention (exposure time) in LNs, important characteristics that motivate the discussion below on the role that duration and concentration of antigen and immune signals in LNs play in driving immunity.

### The Kinetics and Concentration of Antigen Delivery in LNs Can Be Exploited to Enhance Immunity

**Fig. 4:**
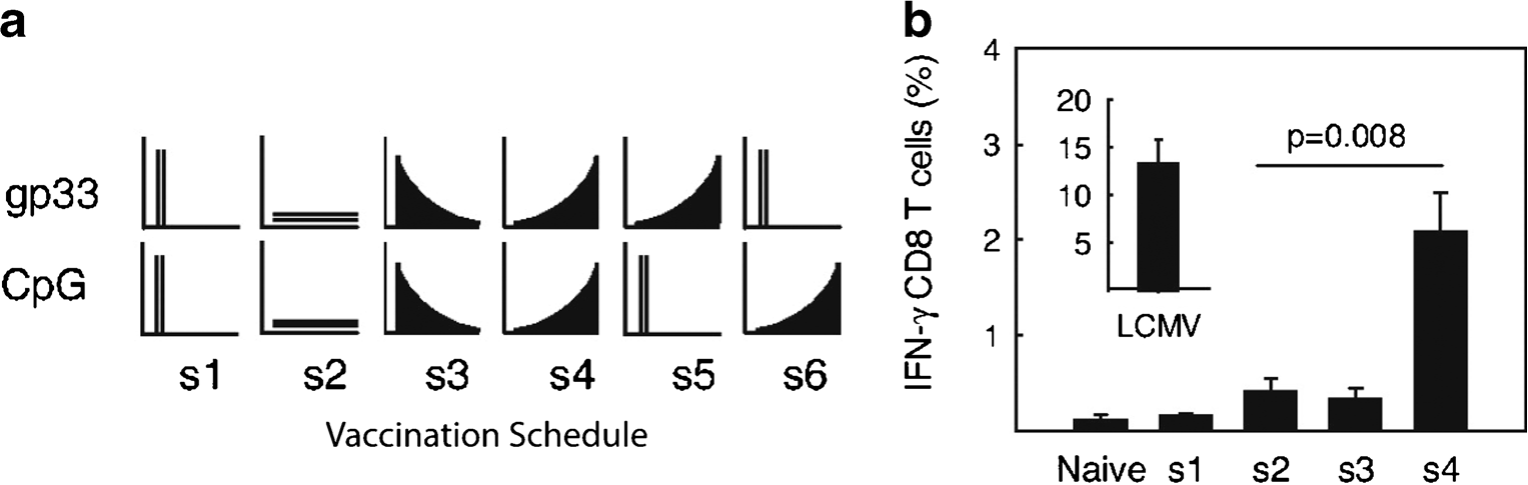
Immune signal kinetics and concentration in LNs controls immunity. **a** Subcutaneous dosing schedule of mice with antigen (gp33) and adjuvant (CpG). **b** IFN-γ production of CD8^+^ T cells 8 days after vaccination with the schedule seen in **a** and restimulation of lymphocytes with gp33. Adapted with permission (40)

An intriguing study by Johansen et al. demonstrated that concentration and duration by which antigens, adjuvants, and immune signals reach LNs are just as important as how efficiently these signals reach LNs. In these studies, mice were immunized subcutaneously with soluble antigen and adjuvant using well-defined doses and injection regimens: (i) one bolus dose, (ii) regularly spaced, equivalent doses, (iii) regular injections with exponentially decreasing doses, or (iv) regular injections with exponentially increasing doses (Fig. 4a). Across all of these regimens, only mice receiving exponentially increasing doses exhibited significantly increased IFN-γ secretion by CD8^+^ T cells (Fig. 4b). Functionally, this effect significantly enhanced antiviral response upon a viral challenge with lymphocytic choriomeningitis virus (40). These results suggest that the persistence and accumulation of antigen and inflammatory signals is important in inducing effective adaptive immune responses. For this reason, the controlled release properties of biomaterials are, and have been, of great interest for vaccine and immunotherapy applications. As illustrated by several of the examples highlighted in the following sections, these approaches hold great potential to direct response, while also reducing the burden on patients through decreasing the number or frequency of injections and treatments.

### Biomaterials Carry Immune Signals to LNs to Promote Changes in LN Structure and Function

**Fig. 5:**
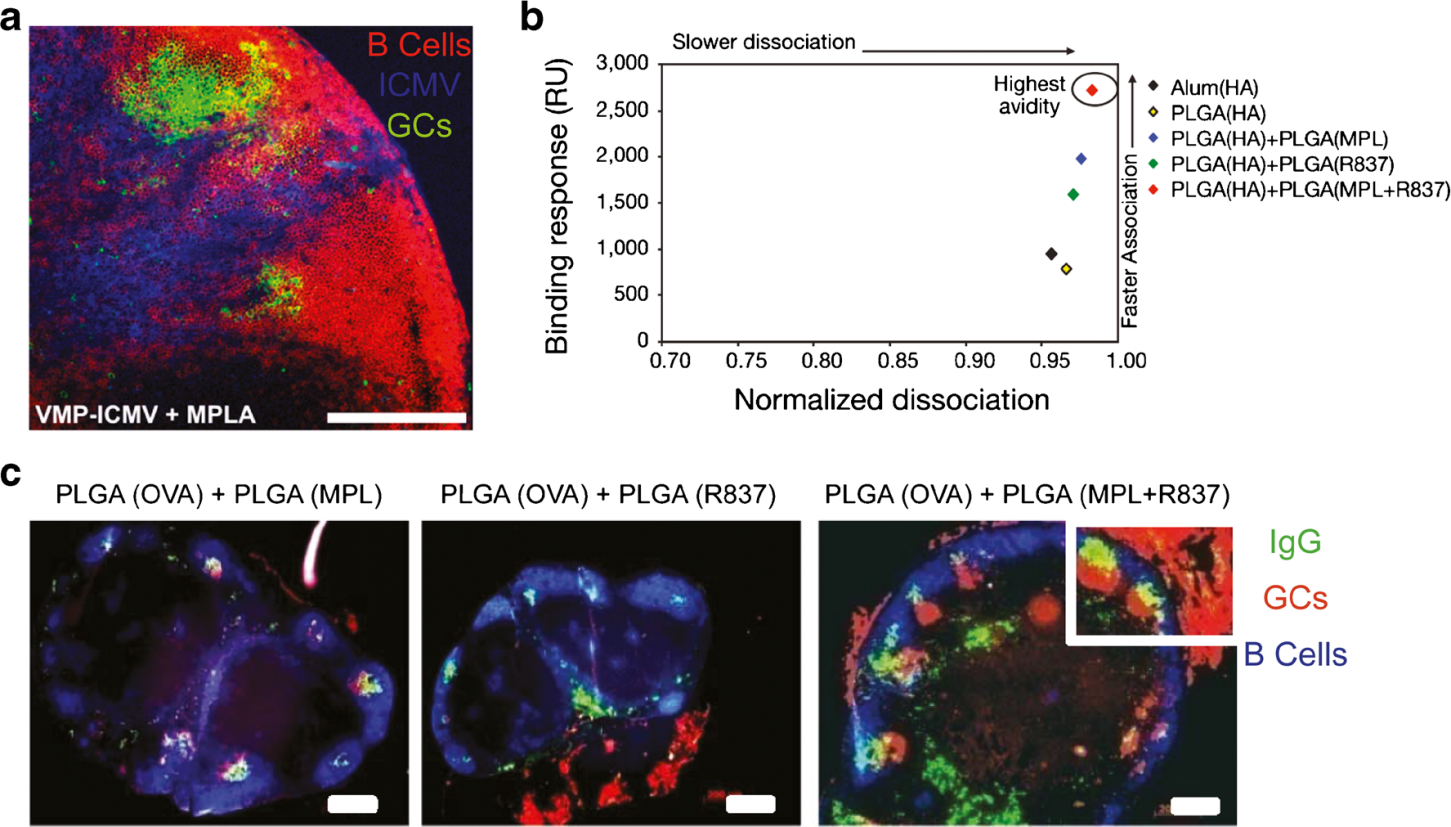
Biomaterial vaccines can enhance GC formation and antibody avidity. **a** Confocal micrograph showing GC formation in draining LN 2 weeks after subcutaneous injection of ICMVs. B cells (*red*, B220), ICMV (*blue*), GC (*green*, GL-7). **b** Hemagglutinin (HA)-binding affinity of serum-derived antibodies from mice immunized with biomaterial vaccine formulations 28 days earlier. **c** 28 days after immunization, draining LNs were excised and stained for GC formation. GC (*red*, GL-7), B cells (*blue*, B220), IgG (*green*). **a** Adapted with permission (41). **b**, **c** Adapted with permission (44)

As alluded to above, NPs, MPs, and other biomaterials offer a unique opportunity to alter local LN structure, and subsequently, systemic immunity by delivering combinations of antigens and adjuvants. This approach has recently been exploited to enhance antibody-mediated immunity by promoting GC creation. These microdomains are required for activation and differentiation of B cells into plasma cells that secrete high-affinity antibodies targeting a specific pathogen. One promising example of this strategy is the synthesis of NPs from interbilayer-crosslinked multilamellar vesicles (ICMVs) (41–43). ICMVs are synthesized by fusing liposomes using divalent cations to form multilamellar vesicles, then crosslinking and PEGylating these structures into 100–300-nm particles. ICMVs have been loaded with a range of vaccine cargos including model antigens (ovalbumin (OVA)), helper T cell peptides, and antigens for malaria and simian immunodeficiency virus (SIV) gag. Following subcutaneous immunization in mice, ICMVs are retained in the draining LN for over 2 weeks and co-localized with macrophages and DCs of the SCS, suggesting that both drainage via lymphatics and transport after APC phagocytosis contribute to ICMVs trafficking to LNs (41). Importantly, immunization with ICMVs increases the number of GCs in the draining LNs compared to soluble vaccine formulations (Fig. 5a) (41). These structures dramatically enhance cell- and antibody-mediated immunity by increasing antigen-specific CD8^+^ T cells and antigen-specific serum antibody levels, respectively. Mice immunized and boosted subcutaneously with ICMV formulations loaded with malaria antigens (VMP-ICMV) generated high levels of malaria-targeted antibodies compared to vaccination with alum as an adjuvant. Strikingly, this effect persisted for more than 400 days after inoculation (41). Since GC formation is integral for high-affinity antibody production and strong humoral immune responses, continued development of materials that promote these structures may be particularly advantageous for parasitic diseases (e.g., malaria) which involve extracellular pathogens that could be bound or neutralized by antibodies. Thus, understanding the link between biomaterial features that are trafficked to particular domains (e.g., SCS and B cell follicles) or support specific interactions or microdomain formation (e.g., GCs) is an important avenue for future research.

Several other approaches using conventional or biomaterial-based vaccines have sought to induce GCs by delivery of multiple adjuvants or TLR agonists. The Pulendran lab has studied the effect of NPs loaded with multiple TLRs in individual particles compared with co-loading these signals in the same particle (44). In these studies, GC formation was strongly dependent on the particle loading scheme used to deliver the TLR agonists. Compared with alum, PLGA NPs loaded with OVA and TLR4 agonist (MPL) or loaded with OVA and TLR7 agonist (R837) increased GC formation in the LNs of mice following subcutaneous injection. Strikingly, mice treated with particles loaded with antigen, along with both TLR4 and TLR7 agonists exhibited a synergistic increase in GCs that also increased the avidity of antibodies (Fig. 5b, c) (44). TLR4 and TLR7 pathways detect bacterial polysaccharides and viral RNA, respectively. Thus, triggering both of these pathways may enhance B cell (TLR4) and T cell (TLR7) activation, as well as generally increase DC functions such as antigen presentation and co-stimulation. The effects of this more robust activation of immune pathways may help inform the design of future materials that contain multiple immune cues.

Recently, vaccines composed of chitosan and heparin have been used to mimic specialized molecules called granules (45). Granules are stable particles secreted by specialized immune cells (mast cells) in response to a range of stimuli that can include pathogen recognition. These particles contain pro-inflammatory cytokines such as TNF-α that promote local inflammation. Following footpad immunization with antigen-loaded NPs designed to mimic granules, NPs localized to the SCS in LNs of mice and increased the number of GCs. The resulting enhancement of antibody-mediated immunity increased the survival of mice during a lethal flu challenge. Interestingly, empty particles without cytokines also caused a modest increase in GCs (45). This effect emphasizes the theme that biomaterials can enhance immunity through targeting, co-delivery, and controlled release of cargo, as well as through stimulatory pathways activated by the structural features of these materials.

### NP Vaccines Can Break Tumor Tolerance Through Local Changes in Tumor-Draining LNs

The examples highlighted thus far share the aim of generating effective immune responses against foreign pathogens. However, another prominent goal of biomaterial-based vaccines and immunotherapies is centered on treating cancer. Cancer cells and the tumor microenvironment exhibit a number of characteristics that hinder the ability of the immune system to fight cancer (46). Notably, effective treatments must generate robust responses against antigens overexpressed on tumors, allow efficient homing of immune cells to tumors, maintain the function of tumor-primed immune cells in the immunosuppressive tumor environment, and generate tumor-specific memory cells that quickly destroy nascent tumor cells to prevent relapse. This is a daunting set of challenges, but combination therapies leveraging biomaterials and the immune system offer many features that could help address these hurdles. Kwong et al. have created liposomes containing both a PEG/CpG lipid that was conjugated via lipid insertion and anti-CD40 antibodies that were added via maleimide chemistry. When these materials are injected into solid tumors, the liposomes drain to nearby LNs and remain in LN sinuses (47). This persistence causes a local adjuvant effect in LNs for at least 48 h that allowed for a majority of LN-resident APCs (DCs and macrophages) to uptake the particles containing CpG, resulting in prolonged survival of mice during tumor challenge compared to PBS controls. Soluble treatments caused a bimodal effect of increasing survival in some mice compared to liposomes and causing earlier death in others, while liposomal delivery reduced systemic toxicity by decreasing adverse side effects such as weight loss and inflammatory cytokines (IL-6) in blood (47).

Stephan et al. approached cancer immunotherapy with biomaterials by modifying the surface of T cells with liposomes or polymeric NPs loaded with cytokines or adjuvants (48). This approach employed biocompatible thiol chemistry to conjugate these materials to cells without altering key T cell functions (e.g., proliferation and antigen recognition). Importantly, tumor-specific CD8^+^ T cells modified with NPs then injected into mice maintained the ability to home to tumor cells, carrying particles and cargo to these sites. This unique approach allowed efficient delivery of cytokine-loaded NPs to melanoma tumors and resulted in rapid proliferation of tumor-specific CD8^+^ T cells in LNs. Mechanistically, T cells conjugated with NPs polarized CD8^+^ T cells toward a central memory phenotype which is more effective at breaking tumor immunosuppression. Thus, mice treated with these tumor-specific T cells modified with NPs eradicated tumors, while untreated mice and mice treated with soluble drugs and T cells all succumbed.

Jeanbart et al. recently exploited preferential drainage of 30 nm poly(propylene sulfide) (PPS) NPs in tumor-draining LNs and distal LNs (i.e., non-draining) for cancer therapy (49). This work revealed that tumor-draining LNs were enlarged compared to distal LNs. DCs and CD8^+^ T cells in tumor-draining LNs also expressed high levels of PD-L1 and PD-1, respectively. Binding of PD-1 on CD8^+^ T cells to the cognate ligand (PD-L1 on APCs) negatively regulates T cell proliferation and pro-inflammatory cytokine secretion, leading to suppression of T cell function. This suppression supports tumor growth during cancer. In these studies, it was shown that while the tumor-draining LN was immunosuppressed, these tissues also contained more antigen-specific T cells, likely due to the proximity to the tumor (49).

Building on this observation, PPS NPs were conjugated with CpG and mixed with antigen-loaded NPs, then injected intradermally into the footpads of mice. NPs containing CpG and tumor antigen (TRP2) or a model antigen (OVA) were injected in the footpad on the same side as tumor induction. This approach resulted in targeting of NPs to the tumor-draining LN. Mice treated in this manner with NPs containing CpG and TRP2 or OVA reduced tumor growth in a melanoma model and in an OVA-expressing lymphoma model. These improvements correlated with an increase in CD8^+^ T cells specific for the corresponding antigens. Targeting the tumor-draining LN also led to a decrease in the number of immunosuppressive cells (e.g., regulatory T cells (T_REGS_)) compared with mice injected on another limb to target the non-tumor-draining LN (49). A related study employed similar NPs co-loaded with CpG and Paclitaxel—a powerful chemotherapeutic—to create a multifunctional cancer therapy (50). Together, these studies highlight the impact of local delivery on efficacy, with particles reaching tumor-draining LNs providing a significantly improved outcome compared with particles targeted to non-draining LNs.

As discussed earlier, once NPs reach LNs, there are also opportunities to direct immunity by delivering multiple signals or by controlling the release of antigens, adjuvants, and other signals. Below, new direct approaches to achieve LN delivery are discussed.

### Intralymphatic Injection Allows Direct Targeting and Local Engineering of the LN Environment

Building on the idea that the kinetics and combinations of immune signals delivered to LNs play an integral role in the development of cell-mediated and antibody-mediated immunity, an intriguing area of fundamental and clinical research has focused on direct injection of vaccines to LNs. In humans, intra-LN (i.LN.) injection generally involves injection of soluble vaccine components to LNs using ultrasound guidance, whereas preclinical studies in mice utilize tracer dyes or surgical procedures to access the LN for injection (51–54). Several important papers from the Kündig lab describe clinical trials demonstrating that i.LN. injection can safely promote tolerance to allergens while dramatically reducing both the cumulative treatment dose and the treatment time (52,55). These fundamental discoveries support the use of i.LN. delivery as a route for generating potent immune response with staggeringly small doses. This approach is particularly attractive for therapeutic applications and has nucleated a number of additional recent and ongoing clinical trials for chronic conditions, cancer, and allergies (40,51,52,56–64). For example, patients immunized i.LN. with a vaccine against grass pollen became tolerized after 3 injections over 8 weeks compared to 54 injections over 3 years when treated with subcutaneous immunization (52). Strikingly, the overall dose needed to evoke this tolerance was more than 1000× lower using i.LN. injections compared with conventional vaccination routes. In a similar study, tolerance to a cat dander allergen was achieved after 3 i.LN. injections and this tolerance persisted for more than 300 days (55). While i.LN. injection is less suited for widespread prophylactic vaccination, this is an intriguing idea for therapeutic vaccines and immunotherapies that rely on delivery of several vaccine components to LNs. However, many of these approaches employ multiple injections or multiple cycles of injections to increase the frequency or duration of exposure to antigen. Thus, coupling i.LN. injection with biomaterials could further enhance the performance of new therapeutic vaccines and immunotherapies while reducing the dose, number, or frequency of injections.

Along these lines, Mohanan et al. tested the delivery of common particle formulations (e.g., liposomes and NPs) along intradermal, intramuscular, subcutaneous, and i.LN. routes. The response to particles laden with OVA antigen or OVA and adjuvants was then assessed by antibody titers and cytokine secretion across injection routes. Formulations injected i.LN. resulted in the highest antigen-specific IgG2a antibody titers regardless of whether or not a TLR agonist (CpG, TLR9) was present in particles. This approach also increased the secretion of INF-γ from splenocytes in the presence and absence of adjuvants (65).

**Fig. 6:**
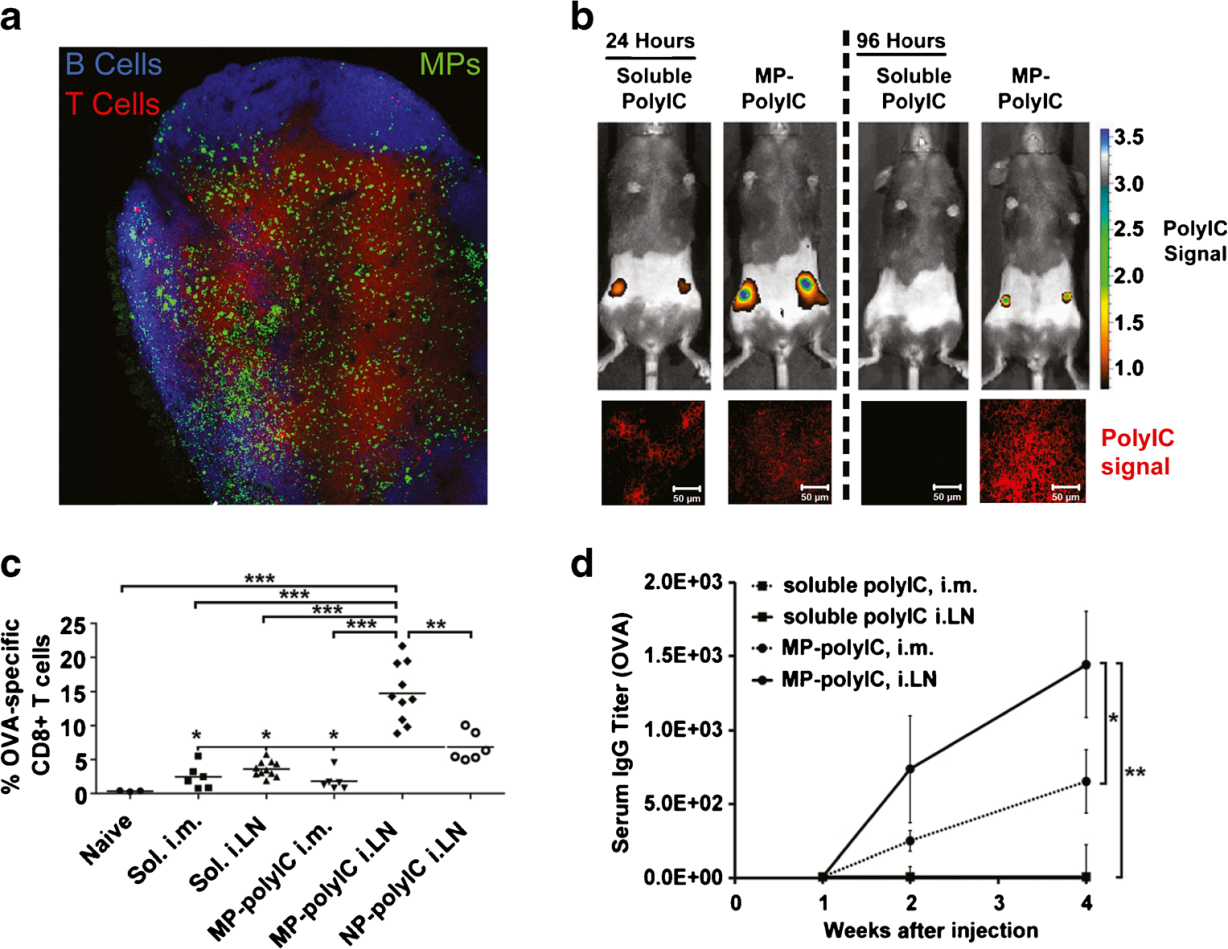
MP depots promote cell-mediated and antibody-mediated immunity after i.LN. injection. **a** Representative image of inguinal LN following i.LN. injection of fluorescent MPs. B cells (*blue*, B220), T cells (*red*, CD3), MPs (*green*). **b** PolyIC signal measured in vivo (*top*) and histological sections of excised LNs (*bottom*) showing PolyIC signal 24 and 96 h after i.LN. injection. **c** OVA-tetramer staining showing percent of blood CD8^+^ cells specific to OVA 7 days after intramuscular or i.LN. injections. **d** OVA-specific IgG serum titers after intramuscular and i.LN. immunization with soluble or MP formulations. Adapted with permission (53)

Jewell et al. developed a non-surgical route for enhancing cell-mediated and antibody-mediated immunity by i.LN. injection of lipid-stabilized polymer particles loaded with adjuvant (53,54). In this study, lipid-coated NPs or MPs loaded with the TLR3 agonist poly(inosinic:cytidylic acid) (polyIC) were injected with OVA antigen into the muscle or into the inguinal LNs of mice (Fig. 6a). MP formulations were retained in the LN as “depots” for at least 4 days, while soluble vaccine formulations were quickly cleared. The increased retention of MPs also controlled release and drove accumulation of polyIC within the LN and in LN-resident APCs, resulting in more enduring activation of DCs (Fig. 6b) (53). These effects potently expanded antigen-specific CD8^+^ T cells circulating in blood 1 week after a single i.LN. injection, an effect that was not observed with soluble vaccine formulations (Fig. 6c). Mice immunized with MPs developed strong antibody responses (Fig. 6d), demonstrating promotion of both cell-mediated and antibody-mediated immune responses. CD8^+^ T cells from MP-immunized mice also exhibited larger, more robust cytokine secretion, and all of these trends persisted for at least 6 weeks without boosting. Interestingly, NPs also increased the number of antigen-specific CD8^+^ T cells and the level of cytokines secreted from these cells compared with soluble vaccines, but at levels lower than those observed in mice vaccinated with MPs (53). This effect was a function of vaccine retention in LNs, with NPs exhibiting a retention time intermediate between the quick-draining soluble formulations and the well-retained MP vaccine depots. Thus, delivery of controlled release depots in LNs mimics the accumulating dosing schemes discussed earlier (Fig. 3a) for soluble vaccines and with i.LN. clinical trials, but with fewer or less frequent injections (40). Such approaches could also help ensure that each component of multifunction vaccines reaches LNs with the correct combinations, doses, or release kinetics.

## BIOMATERIALS CAN ALTER LN FUNCTION TO PROMOTE IMMUNE TOLERANCE

The examples discussed thus far have used biomaterials to promote stimulatory or inflammatory immune responses for vaccination against pathogens or cancer. However, in the past few years, enormous progress has been made in harnessing biomaterials to regulate dysfunctional or unwanted immune reactions. Many of these detrimental reactions occur in autoimmune diseases such as multiple sclerosis, diabetes, rheumatoid arthritis, and lupus, as well as in the rejection of tissue grafts and organ transplants. In multiple sclerosis, for example, myelin—the protein that insulates neurons in the central nervous system (CNS)—is incorrectly recognized as foreign by lymphocytes and antibodies (66–68). This recognition leads to infiltration of these cells and molecules into the CNS, resulting in inflammation and destruction of myelin, and ultimately, neurologic decline. As with the generation of immunity against foreign antigens in healthy individuals, T cells and antibody-producing B cells recognizing self-antigens are also expanded in LNs by APCs presenting these self-molecules. Thus, biomaterials have recently been applied to autoimmunity to stop these reactions by destroying (deletion) or inactivating (anergy) pathogenic cells, or by expanding specialized T_REGS_ which are able to suppress lymphocytes reactive against self-molecules such as myelin. Broadly speaking, these regulatory mechanisms all contribute to immune “tolerance,” a state in which the immune system does not attack, or no longer attacks, a particular peptide, protein, or cell type. One of the greatest challenges facing new therapies for autoimmunity is the induction of self-antigen-specific tolerance that prevents harmful self-reactions without impairing the rest of the immune system. This side effect is a persistent problem with many of the drugs currently used to treat patients with autoimmune disorders: lifelong treatment regimens with broad immunosuppressants are vital to manage disease but cause patients to be immunocompromised. In this section, we will highlight some of the ways in which the interactions between biomaterials and LNs or LN-resident cells are being harnessed to promote tolerance. While the discussion below is focused on the connection between biomaterials and LNs to promote tolerance, several recent reviews provide additional perspective on opportunities to apply biomaterials to autoimmune diseases and tolerance (2,4,69).

### Particles Can Carry Regulatory Signals to LNs to Alter the Interactions of APCs and Lymphocytes

One of the most fundamental ways in which biomaterials can be harnessed to promote tolerance is as a carrier of drugs or other immune signals to LNs or other immunological sites (70–79). In LNs, these cargos can influence the interactions and functions of LN-resident cells in similar ways to those exploited to promote stimulatory responses or immunity. PLGA NPs for example have been loaded with mycophenolic acid (MPA), an immunosuppressant used in transplants (70). Systemic injection of these particles resulted in drainage to the spleen and LNs, where particles were preferentially taken up by macrophages and DCs. During transplant studies, APCs in the LNs of mice treated with particles exhibited elevated levels of PD-L1 (inhibitory ligand) that limited the ability of APCs to prime T cells reactive against antigens expressed on the tissue grafts. Thus, MPA-loaded particles draining to LNs delivered signals that impaired the ability of APCs to expand graft/self-reactive T cells, resulting in tolerance that improved graft survival (70).

The ability of biomaterials to co-deliver multiple cargos has also been exploited to regulate the function of APCs in LNs. In these studies, liposomes were loaded with a self-antigen that is recognized as foreign in mouse models of arthritis, along with a small molecule inhibitor of NF-κB, a protein complex that controls inflammation and that is overexpressed in many chronic inflammatory diseases (i.e., arthritis) (71). Uptake of liposomes by LN-resident APCs reduced NF-κB levels and the proliferation of self-reactive T cells, leading to reduced severity of arthritis. These effects were achieved in part through the expansion of T_REGS_ in mice treated with the liposomes (71). Importantly, the T_REGS_ generated in this study were specific for the self-antigens included in the liposomes, emphasizing the goal stated earlier: inducing tolerance against specific self-antigens, without broad suppression of normal immune functions.

**Fig. 7:**
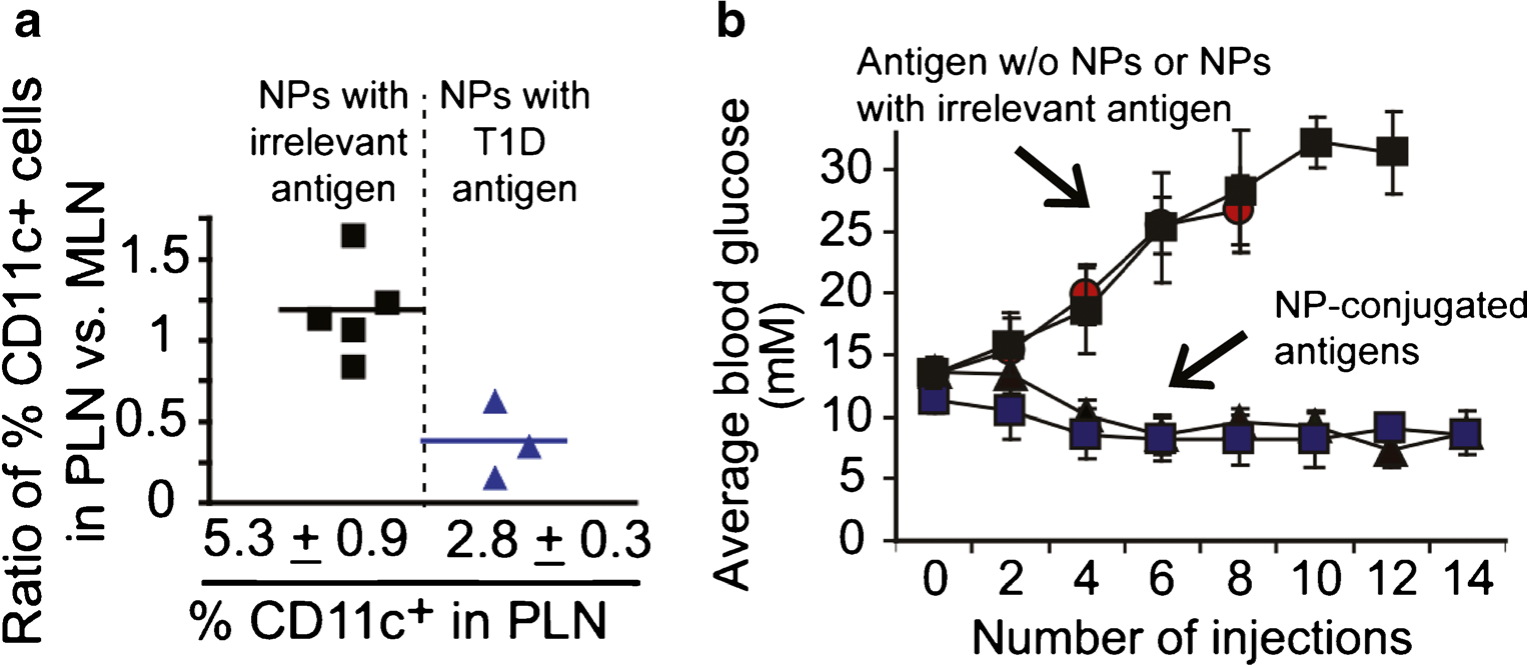
NPs decorated with self-antigen loaded MHC induce DC death and regulate diabetes. **a** NPs decorated with MHC molecules specific to T1D are able to reduce the ratio of CD11c^+^ DCs in the pancreatic LN (PLN) to mesenteric LN (MLN) compared to peptide MHC NPs loaded with an irrelevant antigen. **b** Mice with T1D treated with NPs conjugated with MHC/diabetes antigen complexes maintain normal blood glucose levels compared to treatments with soluble peptide or peptide MHC complexes loaded with irrelevant antigens. Adapted with permission (73)

One intriguing approach being developed to promote antigen-specific tolerance is based on design of NPs decorated with complexes of self-antigen loaded in MHC molecules (73). As discussed earlier, MHCs are the complexes APCs load antigens into for presentation to lymphocytes, along with co-stimulatory signals. Presentation of antigen in MHCs without co-stimulation can cause T cells to become inactive or promote regulatory functions. In this study, iron oxide NPs were functionalized with complexes of MHC and self-antigens associated with disease in type 1 diabetes (T1D), without co-stimulatory signals (73). Treatment of prediabetic or diabetic mice with NPs resulted in expansion of a pool of low avidity (i.e., weakly binding) regulatory T cells in and around LNs near the pancreas—the organ destroyed by self-reactive immune cells in diabetes. These cells suppressed antigen presentation by APCs in these LNs, as well as exhibited direct APC killing in the pancreatic LNs (PLN) compared with LNs remote from the pancreas (MLN) (Fig. 7a) (73). The decrease in APC numbers and activation levels prevented expansion of self-reactive T cells that otherwise could have migrated to and attacked the pancreas. The effects of this treatment were striking, maintaining and restoring control of blood glucose in mouse models of T1D when mice were treated with MHC/NP complexes loaded with T1D antigens, but not when mice were treated with MHC/NP complexes loaded with irrelevant antigens or following injection of soluble T1D antigens (Fig. 7b) (73). Thus, the examples in this section underscore the potential of NPs to deliver drugs and immune signals to alter the interactions of APCs and lymphocytes in LNs during inflammation and autoimmunity.

### Association of Cargo with Biomaterials Can Alter Antigen Trafficking to Promote Tolerance

**Fig. 8:**
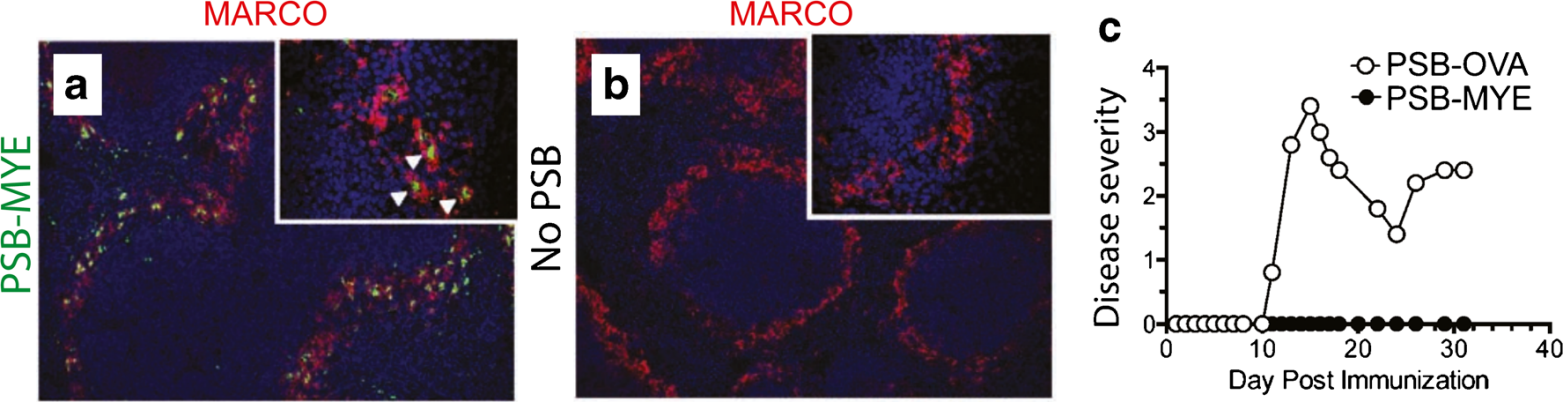
Particles targeting the MARCO receptor induce tolerance against a mouse model of multiple sclerosis. **a** Immunohistochemical staining of spleens following intravenous injection of polystyrene particles conjugated with a myelin peptide (PSB-MYE, *green*) showing co-localization of particles with MARCO (*red*). **b** Immunohistochemical staining as in **a** of spleen after treatment without polystyrene particles (no PSB). **c** Disease severity following immunization with PSB-MYE or polystyrene particles conjugated with an irrelevant peptide (PSB-OVA) showing that antigen specificity is necessary for treatment. Adapted with permission (80)

Another set of approaches recently harnessed to generate tolerance with biomaterials exploits the differences in the mechanisms by which soluble and particulate antigens are trafficked in LNs and spleens. Whereas relatively low-molecular-weight soluble antigens are dispersed throughout SLOs (i.e., LNs, spleen) by the stromal conduits, macrophages and other APCs in the SCS engulf and process larger particles to support presentation of antigenic fragments from these materials. These differences have been exploited to promote antigen-specific tolerance by conjugating 500-nm polystyrene beads (PSB) or PLGA particles with a myelin peptide (MYE)—the self-antigen attacked by the immune system in MS (80,81). Following i.v. injection, antigen-conjugated particles drained to the spleen and were localized to macrophages expressing the scavenger receptor MARCO, whereas free antigen was not (Fig. 8a, b) (80). The MARCO receptor plays an important role in clearing apoptotic cell debris—processes that normally occur without inflammation. Thus, PSB-MYE may support presentation of MYE peptide to APCs in a manner that promotes tolerance (e.g., without co-stimulation). This idea was supported by studies demonstrating that antigen-specific cells in LNs of treated mice exhibited reduced proliferation when challenged with antigen. Treatment with PSB-MYE formulations also effectively treated progressive and recurring models of MS in mice, while treatment with PSBs decorated with irrelevant antigen (PSB-OVA) did not (Fig. 8c). These findings illustrate the antigen-specific nature of tolerance in this system. Mechanistically, this efficacy resulted from increased T_REG_ function, along with reductions in activity of inflammatory T cells (e.g., through anergy/inactivation) (80). These suppressive effects resulted in reduced lymphocyte infiltration to the CNS and decreased inflammatory cytokines (81).

Approaches related to these PSB strategies have also been applied to other targets such as transplantation and inflammatory diseases including colitis, peritonitis, and myocardial infarction (82,83). Of note are studies with inflammatory models using particles exhibiting controlled surface charges but lacking specific antigens. These studies have revealed that inflammatory monocytes engulf negatively charged particles and migrate to the spleen instead of inflammation sites, resulting in apoptosis of these cells and reduced inflammation. Interestingly, neutral particles did not support these therapeutic effects (82). Thus, this strategy could provide a general, non-antigen-specific route for reducing inflammation and further underscores the role of physicochemical properties in determining the types of immune responses biomaterials elicit. Along these same lines, Broere and colleagues have shown that the response to antigen encapsulated in polymers with different carrier structures alters how LN-resident APCs present and interact with helper T cells in LNs (84,85). In particular, PLGA NPs and N-trimethyl chitosan-tri-polyphosphate (TMC-TPP) NPs both increased DC activation and CD4^+^ helper T cell proliferation in LNs, but PLGA promoted regulatory function and reduced hypersensitivity reactions while TMC-TPP stimulated antibody responses. Although the mechanisms of these differences are under investigation, potential contributing factors may include size (which could alter how antigen is trafficked in LNs) or the duration over which these particles release antigen (85).

In addition to solid polymer particles encapsulating or displaying antigen, electrostatically driven condensation of immune signals such as bacterial DNA affects how these components are trafficked within LNs. An approach based on this idea recently revealed that particles formed from bacterial DNA and poly(ethyleneimine) (PEI), a cationic polymer, were rapidly trafficked to the follicular and marginal zones of LNs and the spleen. These complexes stimulated enzyme pathways that promoted DCs and T cells with regulatory characteristics, effects not observed when either PEI or bacterial DNA was administered alone (86). Together, the examples highlighted in this subsection illustrate the potential of designing specific structures or chemistries into biomaterials that can help actively direct how NPs and cargos are trafficked in LNs, as well as to alter the interactions between LN-resident APCs and lymphocytes.

### Biomaterials Can Be Used to Directly Modify Cells to Exploit Regulatory Immune Pathways

In addition to using particles to transport drugs to LNs or change how antigens are processed, several recent approaches have directly modified APCs, lymphocytes, or red blood cells with NPs to promote tolerance or regulate immune response. One group used nanoprecipitation to prepare particles from PEG and poly(lactide) (PLA) conjugated with an immunosuppressant (cyclosporine A (CsA)) (87,88). CsA-loaded particles were phagocytized by DCs incubated with these carriers in vitro, and subsequent injection of these DCs into mice resulted in drainage of the particle-loaded DCs to LNs. These cells locally reduced the number and proliferative capacity of effector T cells in LNs. Other approaches have focused on modification of T cells with, for example, NPs decorated with antibodies specific for the CD4^+^ molecules expressed on helper T cells (89). These particles were loaded with leukemia inhibitory factor (LIF)—a cytokine that can promote the development of T_REGS_—then incubated with donor-reactive cells from the spleens of mice. CD4-targeted LIF-NPs bound CD4^+^ T cells in vitro and transfusion of these cells significantly increased the percentage of T_REGS_ in LNs over 5 days. Thus, modifying T cells with regulatory immune signals can serve as a route to deliver cues to LNs that alter how T cells develop during antigen presentation. This goal of controlling T cell differentiation shares similarities with the work of Stephan et al., though their approach aimed to generate immunostimulatory responses for cancer therapy by modifying T cells, as discussed earlier (48).

**Fig. 9:**
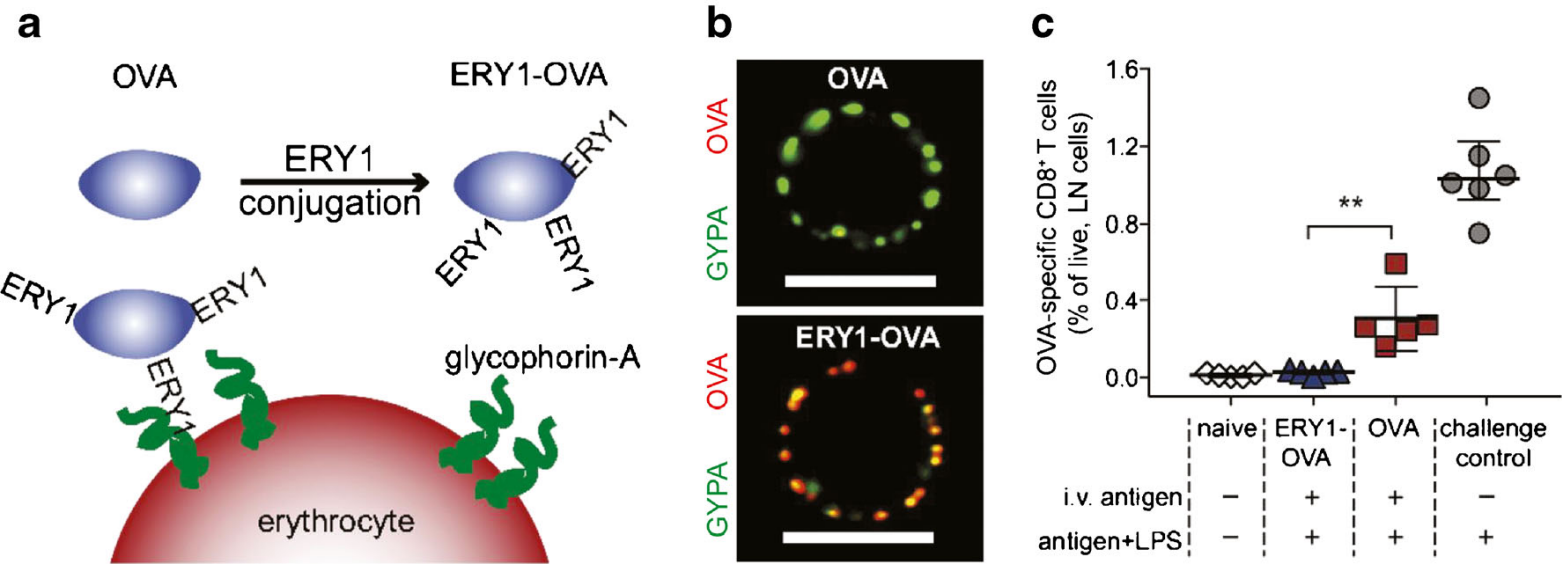
Erythrocyte decorating with NPs leads to immune tolerance. **a** Graphic depicting the conjugation of OVA-NPs to the surface of erythrocytes via ERY1 binding of glycophorin-A. **b** Confocal images showing an erythrocyte treated with soluble OVA (*top*) and with OVA-NP (*bottom*). OVA (*red*) is colocalized with glycophorin-A (GYPA, *green*). **c** Percentage of antigen-specific CD8^+^ T cells within the draining LN 4 days after immunization with erythrocytes labeled with OVA-NPs (ERY1-OVA) or soluble OVA. Adapted with permission (91)

Significant fractions of erythrocytes (i.e., red blood cells) are rapidly produced and destroyed on a daily basis in healthy individuals. In these cases, cell destruction occurs through a non-pathogenic mechanism of cell death, apoptosis (90). This mechanism does not induce pro-inflammatory immune responses against these cells owing to natural regulatory mechanisms that clear self-antigens without co-stimulation or due to activation of suppressive pathways triggered by apoptotic cell debris. This natural tolerance pathway has recently been exploited to generate antigen-specific tolerance against model antigens in disease models of T1D (91). To conduct these studies, a target antigen (OVA) was conjugated to a peptide (ERY1) that binds glycophorin-A molecules (GYPA) expressed on the surface of erythrocytes (Fig. 9a). Ex vivo incubation of ERY1-OVA with mouse erythrocytes resulted in efficient labeling of these cells with the target antigen (Fig. 9b, bottom), whereas incubation of unmodified OVA with red blood cells did not result in cell labeling (Fig. 9b, top). Intravenous injection of cargo-modified ERY1 (e.g., OVA and fluorescent dye) quickly labeled circulating erythrocytes and led to increased trafficking to the spleen and uptake by resident APCs. Mice treated with ERY1-OVA after injection of OVA-responsive transgenic T cells exhibited reduced proliferation of these cells in LNs and in the secretion of inflammatory cytokines (e.g., IFN-γ) (Fig. 9c). This idea was also exploited to protect mice from T1D by stimulating proliferation and rapid deletion of self-reactive CD4^+^ T cells in LNs when mice were treated with ERY1 conjugated to T1D antigens, but not when soluble T1D antigens were administered. Together, these approaches demonstrate that a diverse set of cell modification approaches can alter how APCs and T cells function, as well as harness natural apoptotic clearance and tolerance mechanisms to regulate or redirect inflammatory immune reactions.

## CONCLUSIONS AND LOOKING FORWARD

Biomaterials have already demonstrated great potential in the field of immunology. In coming years, continuing to improve our understanding of how material properties impact molecular signaling will remain an important issue, as this knowledge will lead to more rational design of vaccines. Employing biomaterials as tools to study new fundamental questions will also provide new opportunities to inform vaccine and immunotherapy design. An exciting avenue of research centers on engineering biomaterials with specific properties that allow for precise interactions with LNs and LN-resident cell populations that effectively modify the structure of these tissues. Some of these questions may also focus on understanding how the kinetics and combinations of immune signals in LNs impact stromal function (e.g., FRC network, laminins), and if these changes can be induced or exploited to direct immunity. Another interesting approach on the horizon is the design of artificial LNs or SLOs that could locally recapitulate the functions of these tissues, perhaps eliminating the targeting challenges facing many vaccines and treatments. Biomaterials offer unique opportunities to address each of these areas, and the answers to these questions will continue to push the forefront of what may become possible in modulating immune function.
